# Anatase TiO_2_ nanotube powder film with high crystallinity for enhanced photocatalytic performance

**DOI:** 10.1186/s11671-015-0814-6

**Published:** 2015-03-04

**Authors:** Jia Lin, Xiaolin Liu, Shu Zhu, Yongsheng Liu, Xianfeng Chen

**Affiliations:** Department of Physics, Shanghai University of Electric Power, 2103 Pingliang Road, Shanghai, 200090 China; Department of Physics and Astronomy, Shanghai Jiao Tong University, 800 Dongchuan Road, Shanghai, 200240 China

**Keywords:** Titanium dioxide, Nanotube, Anodization, Crystallization, Photocatalysis

## Abstract

We report on the synthesis of TiO_2_ nanotube (NT) powders using anodic oxidation and ultrasonication. Compared to free-standing NT array films, the powder-type NTs can be easily fabricated in a cost-effective way. Particularly, without the substrate effect arising from underlying Ti metals, highly crystallized NT powders with intact tube structures and pure anatase phase can be obtained using high-temperature heat treatment. The application of NTs with different crystallinity for the photocatalytic decomposition of methylene blue (MB) was then demonstrated. The results showed that with increasing annealing temperature, the photocatalytic decomposition rate was gradually enhanced, and the NT powder electrode annealed at 650°C showed the highest photoactivity. Compared to typical NTs annealed at 450°C, the rate constant increased by 2.7-fold, although the surface area was 21% lower. These findings indicate that the better photocatalytic activity was due to the significantly improved crystallinity of anatase anodic NTs in powder form, resulting in a low density of crystalline defects. This simple and efficient approach is applicable for scaled-up water purification and other light utilization applications.

**Electronic supplementary material:**

The online version of this article (doi:10.1186/s11671-015-0814-6) contains supplementary material, which is available to authorized users.

## Background

In the past three decades, titanium dioxide (TiO_2_) and its nanocomposites have been widely investigated as promising photocatalysts [1-4]. Among various TiO_2_ nanostructures, TiO_2_ nanotube (NT) arrays synthesized by a simple electrochemical anodization provide the advantages of adjustable structures, high surface area, and excellent charge transport properties. Various applications such as photocatalytic decomposition, water splitting, photovoltaics, batteries, and sensors have been explored. TiO_2_ NTs have been found to have better photocatalytic properties of organic pollutants compared to commonly used TiO_2_ nanoparticles, although the NT surface area is much smaller [5-7]. This is due to the excellent light trapping, enhanced electron-hole separation, and much slower deactivation of NTs during the photocatalytic reaction [8]. To further enhance the photocatalytic activity of NTs, various strategies have been used such as the improvement of morphology, crystal structure, and surface area [9,10], introduction of heterogeneous structures by decoration [11,12], and metal or non-metal doping [13,14]. Among these, the enhancement of NT crystallinity has been considered as an important and widely investigated approach to achieve better photocatalytic performances.

NT crystallinity can be simply enhanced by increasing the annealing temperature. More importantly, the stability of structures and geometries has to be considered during annealing. As to aligned NT array, the retention of its porous structure during annealing is essential to preserve its large specific surface area, which is desired for photocatalytic reaction. However, for NT arrays fabricated on metallic Ti foils, the substrate effect would result in crystallite growth in the tube walls during the high-temperature annealing process, leading to thicker tube walls and a significant decrease of the surface area. Therefore, the discussion of optimal annealing temperature and crystallinity is difficult. The highest photodegradation efficiency has been reported using NT films annealed at different temperatures such as 450°C [15], 500°C [16], 550°C [17], and 600°C [18]. These earlier studies have concluded that the tubular structure was stable at or below the optimal temperatures. On the other hand, at higher temperatures, the NT structure collapses and undergoes deformation, dramatically decreasing the surface area and thus photocatalytic activity. Another disadvantage caused by the substrate effect is the phase instability of NTs under high-temperature treatment. TiO_2_ NTs with a stable anatase phase have excellent performance in photocatalytic and photoelectrochemical reactions [17,19-21]. However, after annealing of the as-formed NTs on their metallic substrate above approximately 550°C, a crystal phase transformation from anatase to rutile developed [15,22], which would play a role in lowering the photocatalytic performance.

The synthesis of free-standing NT films is a promising way to generate NTs without the presence of a Ti metal substrate, resulting in both structure and phase stability up to a high temperature of approximately 600°C [23] or 700°C [24], although the fabrication process is tedious and inefficient. By rapid breakdown anodization [25,26], NT bundles in powder form can be prepared. However, these bundles featured broken tubes and also contained impurities of remnants from the anodization electrolyte. Therefore, the bundles maintained stable phase and structure specifically at temperature <450°C. Therefore, the development of alternative methods is warranted. In the present study, we evaluated TiO_2_ NTs in powder form by dispersing as-anodized NT arrays. The morphology, structure, and crystal phase composition of the synthesized NT powders were characterized. The photocatalytic property of the powders annealed at different temperatures was also investigated to achieve optimized photocatalytic activity.

## Methods

### Preparation of the NT powders

The preparation details of anodic NT arrays were similar to our previous reports [27]. Self-organized TiO_2_ NTs were grown on a Ti substrate (10 × 10 cm, 0.89 mm thickness, 99.7% purity, Alfa Aesar, Ward Hill, MA, USA). Then, the as-anodized irregular surface oxide layer was stripped off the Ti substrate by ultrasonication in deionized (DI) water. The cleaned Ti foil was anodized again for 2 h to grow aligned NT arrays in the same electrolyte. After the two-step anodization, ultrasonication in ethanol or DI water was carried out for 5 to 10 min to disperse the obtained oxide tube layer. Then, the Ti foil was reused for anodization formation of NTs. The above process was repeated until the Ti foil was completely consumed. The NT powder was collected by centrifugation and dried in a vacuum drying oven at 120°C overnight. The powder product was heat-treated at 450°C, 550°C, 650°C, and 750°C for 2 h in air to crystallize the NTs.

### Characterization of the NT powders

The morphology of the resulting NT powders was characterized by a field emission scanning electron microscope (FE-SEM; Sirion 200, FEI, Hillsboro, OR, USA). The relative Brunauer-Emmett-Teller (BET) surface area was evaluated by adsorption-desorption isotherms using nitrogen gas at 77 K (ASAP 2010, Micromeritics, Norcross, GA, USA). Samples were degassed at 200°C for 4 h under high vacuum prior to measurement. The pore size distribution was analyzed by Barrett-Joyner-Halenda (BJH) adsorption differential pore volume. TiO_2_ paste was prepared by adding of the powder (20 wt%) to a mixture of isopropanol:n-butyl alcohol = 1:4 (*v*/*v*) solution. Thereafter, the paste was mixed under magnetic stirring for 24 h. The paste was deposited on fluorine-doped tin oxide (FTO) glasses by using a doctor blade technique and then air-dried, forming a porous NT film. The film was reinforced by annealing again at 450°C. The thickness of the NT layer was approximately 10 μm, which was controlled by tapes (Scotch Magic Tape, 3 M, St. Paul, MN, USA). The crystal structures were verified by X-ray diffraction (XRD; Cu K_α_ radiation, Rigaku 9KW SmartLab, Rigaku, Tokyo, Japan) patterns.

### Photocatalytic measurements

The photocatalytic properties of the samples annealed at four different temperatures were evaluated by the photodecomposition of methylene blue (MB). An NT-coated substrate with an active area of 9 cm^2^ was immersed in 100 mL of MB aqueous solution (initial concentration: 10 mg/L) for 1 h in the dark to reach the adsorption-desorption equilibrium at the TiO_2_ surface. Ultraviolet (UV) light (*λ* = 365 nm) was provided by a UV lamp assembly (8 W × 3) with the distance between lamps and the photocatalyst film of approximately 15 cm (irradiation intensity of 5 mW/cm^2^). All the samples were tested under the same condition. The absorbance of the MB solution was measured using a UV-vis/NIR spectrophotometer (UV-3600, Shimadzu, Kyoto, Japan) at a wavelength of 664 nm to determine variations in MB concentration with the UV irradiation time.

## Results and discussion

**Figure 1:**
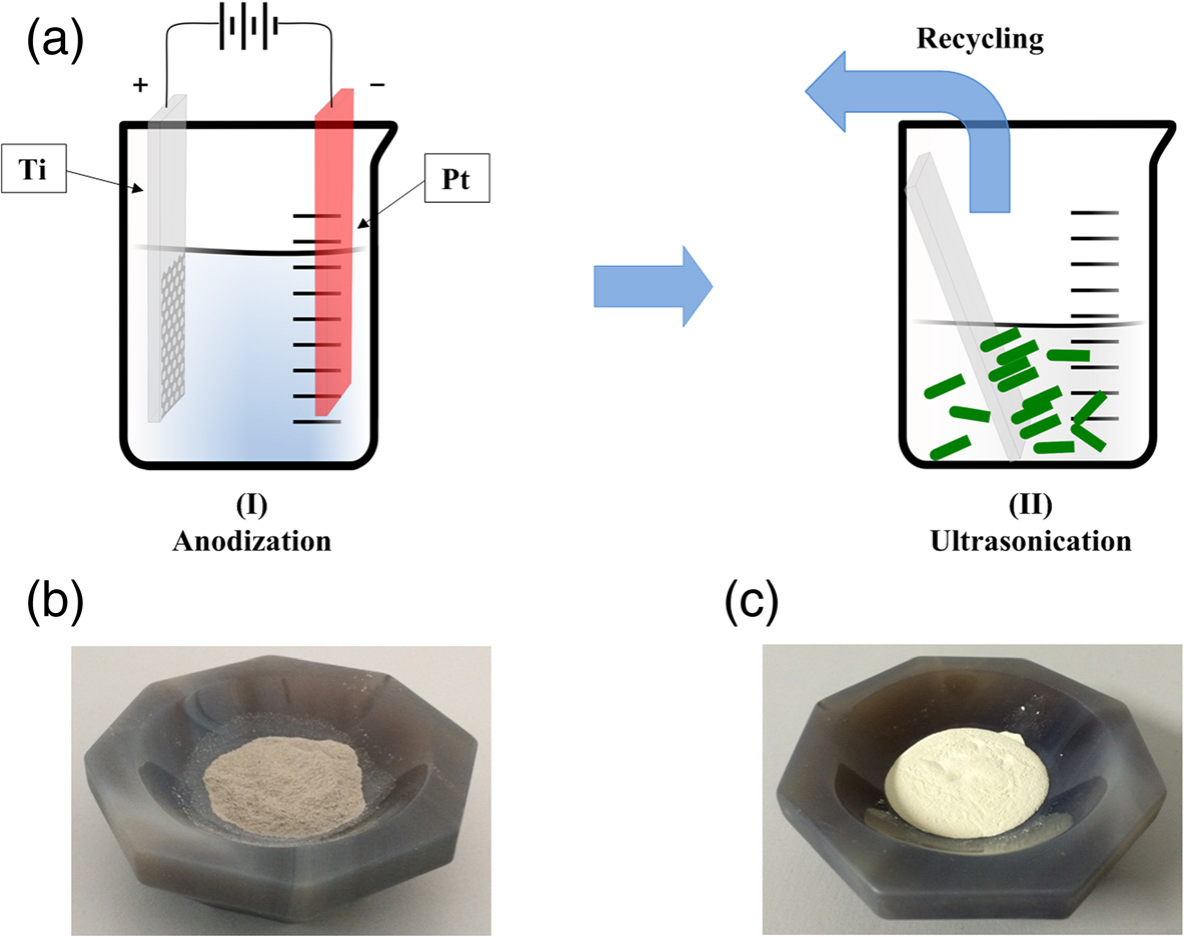
**Schematic and images of the NT powders. (a)** Schematic illustration of the fabrication process of NT powders, including (I) anodization of Ti foil to produce NT arrays and (II) ultrasonication in ethanol to disperse the as-formed NTs. **(b)** The amorphous NT powder obtained by collecting the dispersed NTs. **(c)** Crystallized NT powder after annealing.

It has been previously reported that for NTs grown on a Ti substrate, direct annealing to crystallinity results in severe substrate effects [24,28], resulting in the destruction of tubes and a largely decreased surface area. Herein, by ultrasonication in ethanol, the as-anodized NTs could be detached from the Ti substrate prior to usage (Figure 1a), and thus, the influence of the Ti substrate was successfully eliminated. After the removal of surface NTs, the Ti foil could be reused until the foil was completely consumed. The reaction yield each time (approximately 2 h) per cm^2^ area of the Ti foil was approximately 0.01 g. The as-prepared NT powder was amorphous and showed a gray color (Figure 1b). After the crystallization at 450°C, the color of the NT powder changed to white (Figure 1c). Compared to NT arrays, dispersed NT powders showed high quality, and thus, the dimension and phase of NTs could be stabilized during high-temperature crystallization, as will be discussed in the next section.

**Figure 2:**
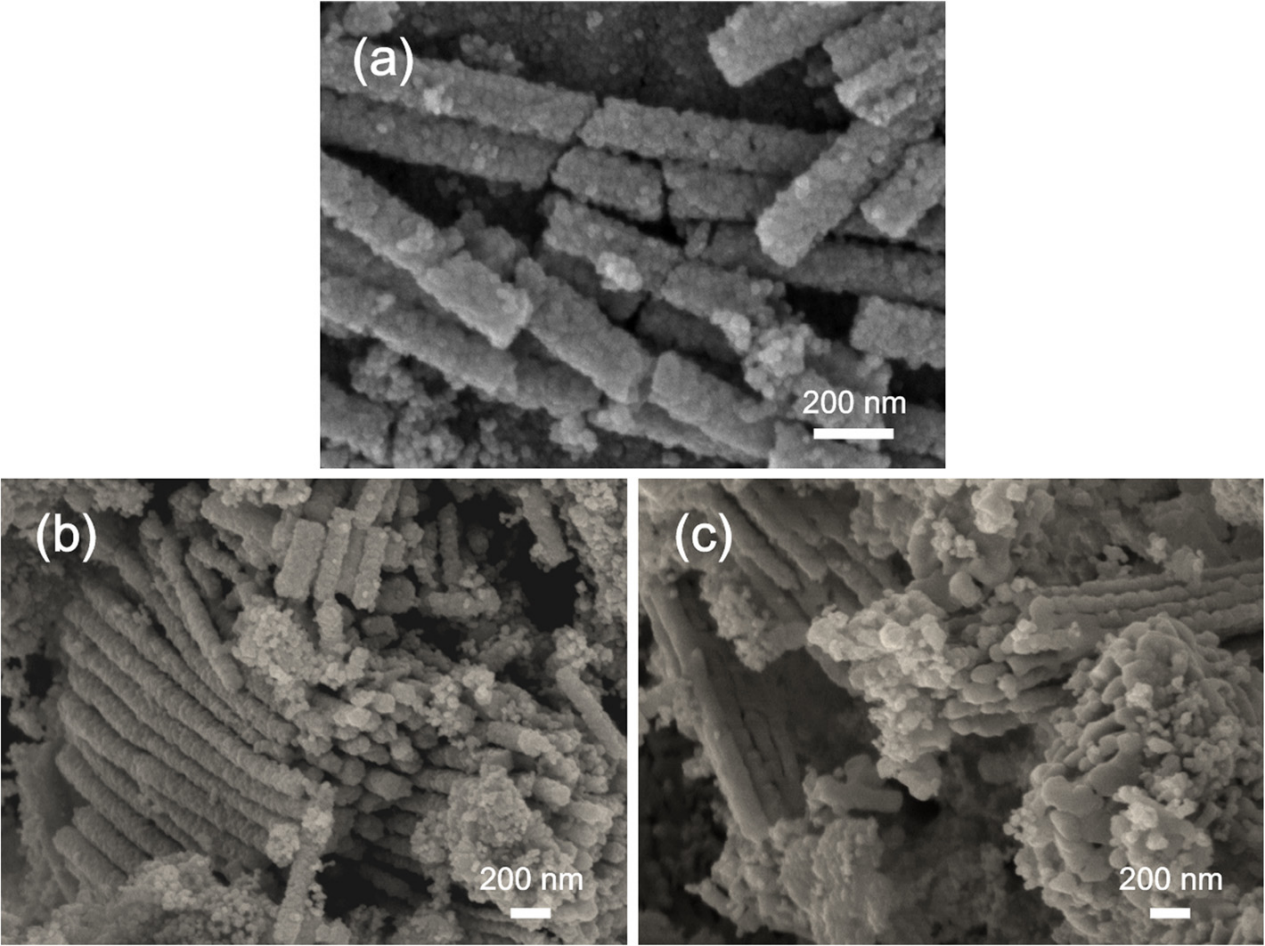
**SEM images of the NT powders. (a)** SEM morphology of the as-prepared NT powders. SEM images of the NT powders annealed at **(b)** 450°C and **(c)** 750°C.

The SEM images of the as-formed NT powders showed a tubular morphology. The tube arrays were separated into isolated tubes, and the entire tube was broken into several segments. Figure 2a shows that the tube wall consisted of TiO_2_ nanocrystallites. After annealing at 450°C, loosely packed tube agglomerates were observed (Figure 2b). By further increasing the crystallization temperature of the NT powders, the tubular structure was well preserved, with slight structure changes. The SEM image of the samples annealed at 750°C is shown in Figure 2c. However, when NTs were dispersed in DI water, changes in structural integrity were more apparent. Heat treatment at 750°C resulted in the collapse of the NT structure (Additional file 1: Figure S1). The tubes were severely sintered and consolidated, and the tubular architecture gradually disappeared. The changes might probably be due to the water-induced tube wall modification as has been previously reported, leading to a hybrid structure [29]. In addition, to recycle NT powder as a photocatalyst, it was immobilized onto an FTO substrate. From the cross-sectional view in Additional file 1: Figure S2, a uniform, dense, and randomly packed TiO_2_ film was tightly connected to the substrate, promising for use in photocatalytic reactions.

**Figure 3:**
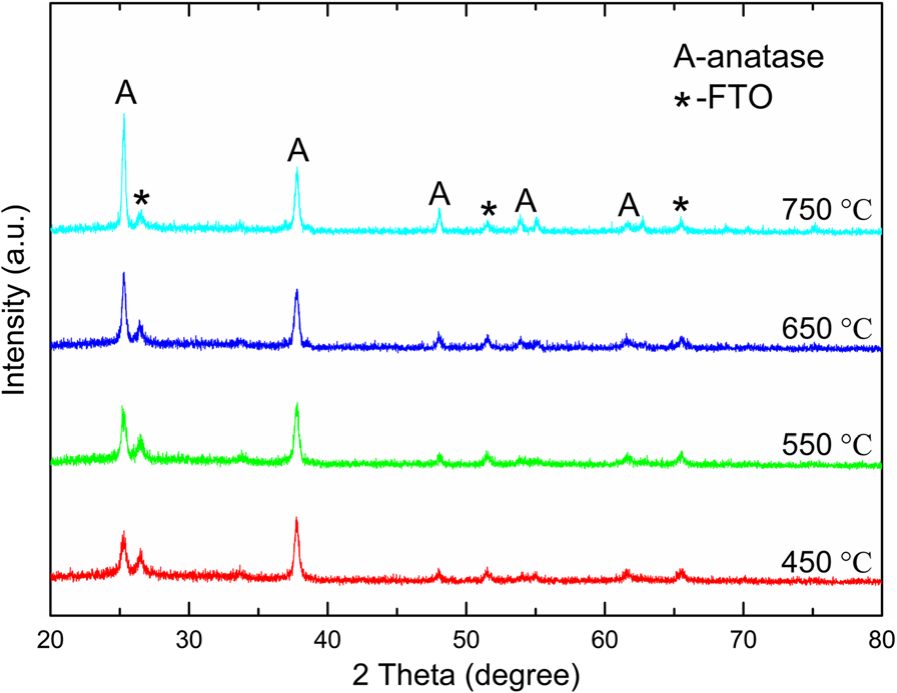
**Crystal phase characterization.** XRD patterns of the NT powders annealed at different temperatures ranging from 450°C to 750°C.

The effect of annealing temperature on crystal structure is then examined. For NTs on the Ti substrate, rutile developed from the oxidization of underlying Ti metals and was gradually transmitted to the upper tube region. Therefore, in the absence of the Ti metal substrate, no rutile phase was observed up to 750°C, and the anatase phase was maintained (Figure 3). This indicates the NT structure and crystal phase of powders were highly stable. The anatase (101) diffraction peak was more prominent with increasing annealing temperatures, indicating an enhanced crystallinity of the anatase phase. However, the unstable hybrid structure that was dispersed in water could be destroyed during high-temperature annealing, showing a phase transition from anatase to rutile at 650°C, as indicated by the XRD patterns shown in Additional file 1: Figure S3. Furthermore, at 750°C, most of the anatase have been converted to the rutile phase.

**Figure 4:**
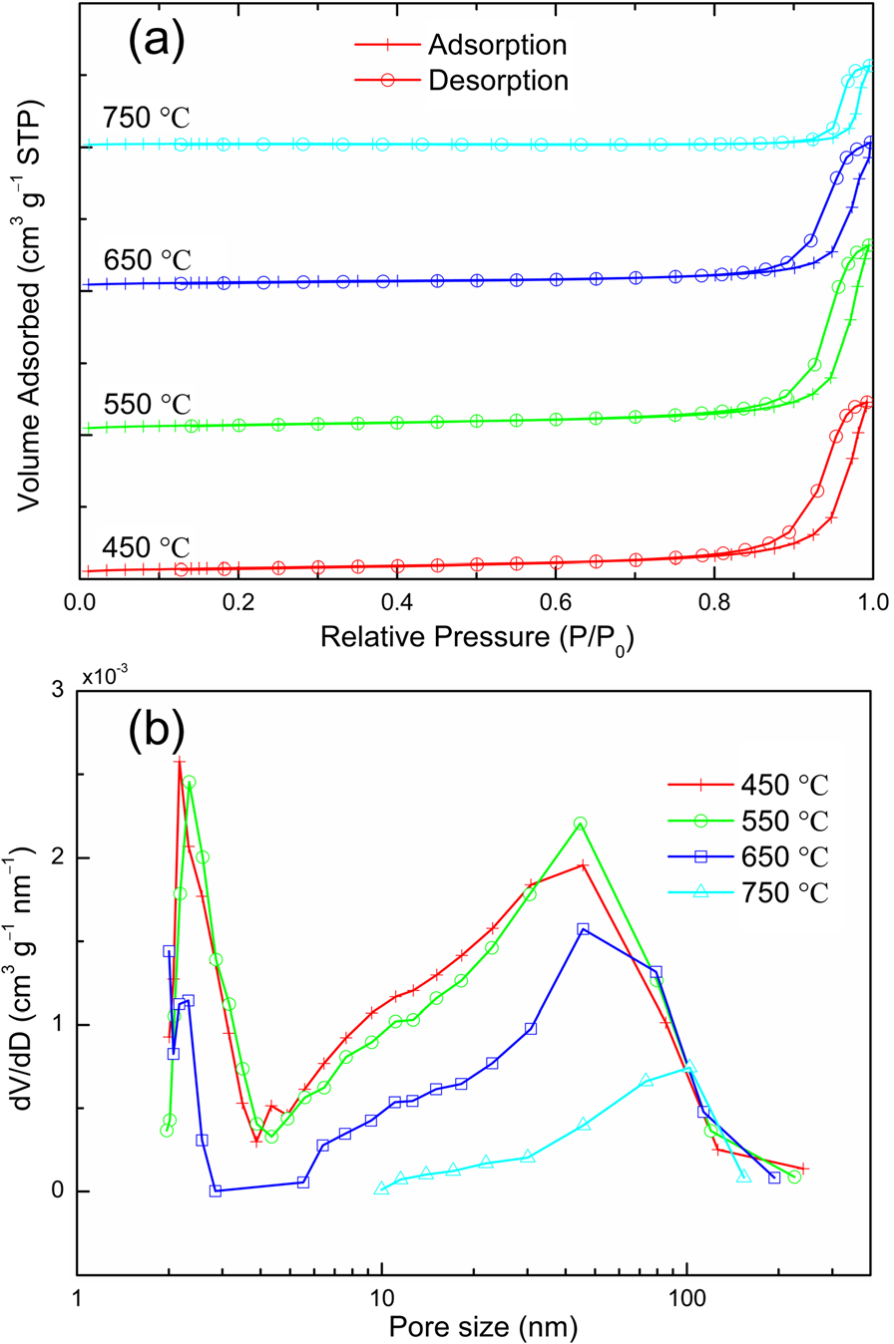
**Nitrogen sorption isotherms and pore size. (a)** Nitrogen adsorption-desorption isotherms of the NT powders annealed at various temperatures. **(b)** Corresponding pore size distribution.

The amount of pollutant molecules adsorbed onto the TiO_2_ surface is an important factor that influences photocatalytic activity. For a larger surface area, the amount of adsorption can be high, resulting in improved photocatalytic activity. For example, when the NT film thickness increased, the rate constant of photocatalytic reaction was significantly improved [5,7,30]. Thus, the surface area and pore size distribution of NT powders were investigated through nitrogen sorption analysis. Figure 4a shows the nitrogen adsorption-desorption isotherms of the NT powders annealed at various temperatures. The BET surface area shows a decreasing tendency with increasing annealing temperatures. The specific surface areas of the NTs annealed at 450°C, 550°C, 650°C, and 750°C were 26.7, 24.9, 21.0, and 8.0 m^2^/g, respectively. The NT powders possessed a very limited surface decrease up to 650°C, indicating that crystallite growth and aggregation of the NT powder were restricted to the tube walls. However, at 750°C, although the anatase phase was well preserved as previously described, due to the enlargement of TiO_2_ nanocrystallites, the surface area presented a sharp decrease, which in turn reduced the photocatalytic degradation rate. Figure 4b shows a bimodal BJH average pore size distribution with two peaks, which was analyzed based on adsorption branch. The average pore size increased with higher temperatures, from 16.22 to 38.98 nm. The larger pores were formed during the sintering of the nanocrystallites in the NTs at higher temperatures. Therefore, a few pores were present inside the film, and a reduction in internal surface area was observed. The peaks located at the value of approximately 2 nm corresponded to the mesopores in the tube walls.

**Figure 5:**
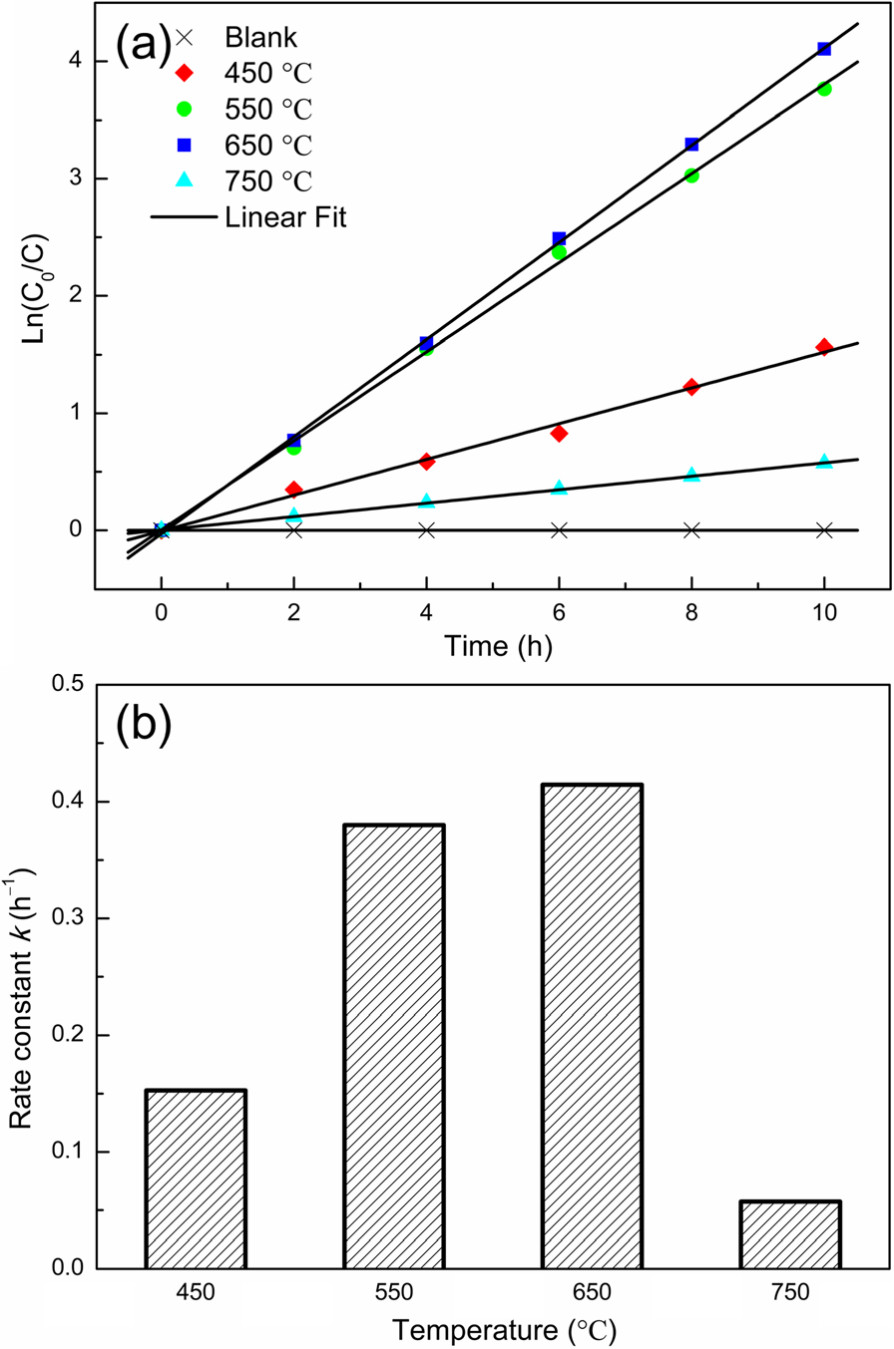
**Photocatalytic performance and rate constant. (a)** Time-dependent MB concentration showing the photocatalytic decomposition kinetic behavior of the NT powders obtained at various annealing temperatures of 450°C, 550°C, 650°C, and 750°C. **(b)** The photocatalytic rate constant.

To investigate the photodegradation of MB solution by the annealed NT powder electrodes, variations in MB concentration as a function of illumination time were monitored (Figure 5a). In the absence of the NT photocatalyst, the MB solution showed negligible self-degradation. MB molecules decomposed faster with increasing annealing temperatures within the specific range of 450°C to 650°C, indicating that NTs of higher crystallization possessed better photocatalytic activity. In addition, the 750°C sample showed a lower photocatalytic activity. The linear plot in Figure 5a indicates that the photocatalysis process followed a quasi-first-order kinetics and thereby can be presented as ln(*C*_0_/*C*) = ln(*A*_0_/*A*) = *kt*. Here, *C*_0_ is the initial MB concentration, and *C* is the concentration after irradiation for time *t. A*_0_ and *A* are the corresponding absorbances. *k* is a first-order rate kinetic constant, which well represents the photocatalytic activity. A comparison of the rate constant values at different temperatures is presented in Figure 5b.

For the 550°C and 650°C samples, after photocatalytic reaction, the aqueous solutions were almost completely decolorized, showing complete degradation of MB. Although the 650°C sample presented a relatively small surface area, its photocatalytic activity was the highest, with the rate constant of 0.414/h. During the photocatalytic oxidation, the photogenerated electrons and holes either recombined inside TiO_2_ nanocrystallites or reacted with adsorbed species on the TiO_2_ surface. The crystallinity of NT powders gradually improved with increasing annealing temperatures, resulting in (a) decreased density of residual elements and thus diminished crystalline defects in anodic tubes [31], which in turn led to lower electron-hole recombination probability in bulk, and (b) effective diffusion of electrons and holes to adsorbed reactants on the TiO_2_ surface to participate in the decomposition reaction. Both factors lead to a more efficient electron-hole separation and thus enhanced photocatalytic activity. On the other hand, for the 750°C sample, a significantly smaller surface area and, in turn, a lower photocatalytic activity were observed.

## Conclusions

We present a simple and cost-effective approach for the growth, dispersion, annealing, and immobilization of TiO_2_ NT powders. The structure and crystal phase of the NT powders at low and high temperatures were investigated. The NTs in powder form showed improved structure and crystal phase stability under high-temperature treatment compared to the conventional NTs on a Ti substrate. By increasing the temperature from 450°C to 650°C, the highly crystallized NT photocatalyst showed a significantly enhanced photocatalytic activity, mainly due to the low density of defects. Furthermore, an increase in temperature from 650°C to 750°C led to worse photocatalytic activity due to the aggregation of NTs and thus a largely diminished surface. Further utilization of high-crystallized NTs in photoelectrocatalytic degradation is currently in progress.

## Additional file

Additional file 1:
**Supporting information.** The file contains Figures S1 to S3.
