# Supplementary material for: Anatase TiO2 nanotube powder film with high crystallinity for enhanced photocatalytic performance
Source: Nanoscale Res Lett. 2015 Mar 4;10:110. doi: 10.1186/s11671-015-0814-6 (PMC4385124; doi:10.1186/s11671-015-0814-6)
Supplement: Additional file 1: — Supporting information. The file contains Figures S1 to S3. [file 11671_2015_814_MOESM1_ESM.doc]

**Supporting Information**

**Anatase TiO_2_ nanotube-powder film with high crystallinity for enhanced photocatalytic performance**

Jia Lin^1^, Xiaolin Liu^2^, Shu Zhu^2^,Yongsheng Liu^1*^ and Xianfeng Chen^1, 2*^

^1^ Department of Physics, Shanghai University of Electric Power, 2103 Pingliang Road, Shanghai 200090, China

^2^ Department of Physics and Astronomy, Shanghai Jiao Tong University, 800 Dongchuan Road, Shanghai 200240, China

*Corresponding author: ysliu@shiep.edu.cn, xfchen@sjtu.edu.cn


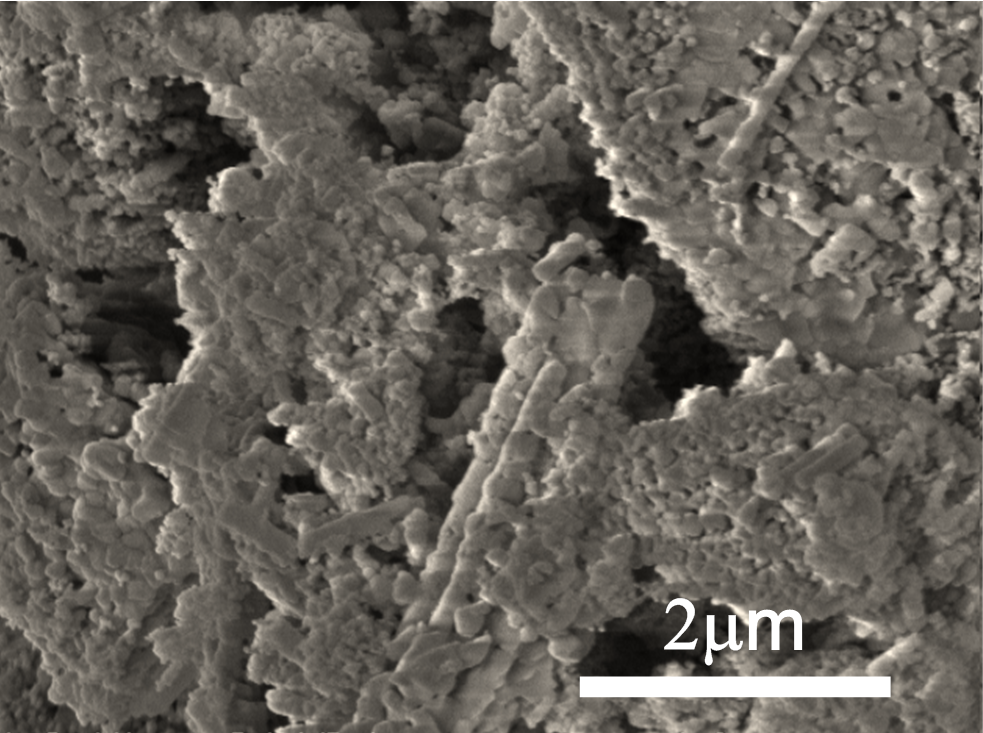


**Figure S1** The SEM image of the collapsed tube powders annealed at 750 °C.


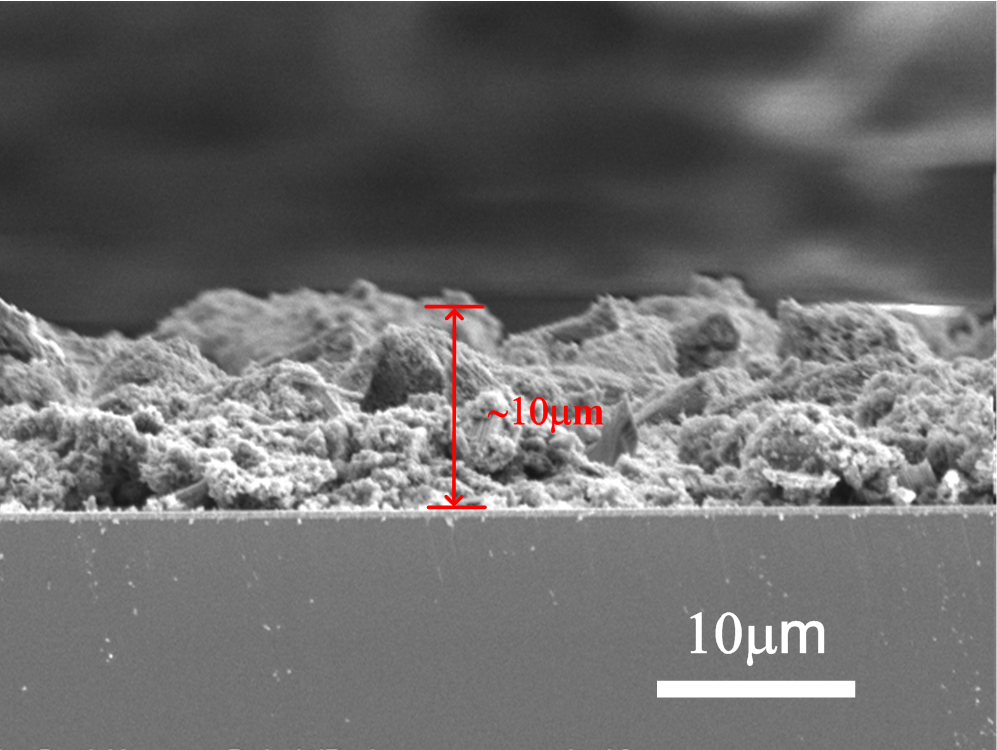


**Figure S2** The SEM image of ~10 μm thick layer of NT powders annealed at 650 °C by doctor blade coating on an FTO substrate.

Compared with NT powder suspensions, tube powders compacted to NT films on supporting substrate can be easily separated from pollutant solutions and reused. This recyclable characteristic is crucial for practical applications.


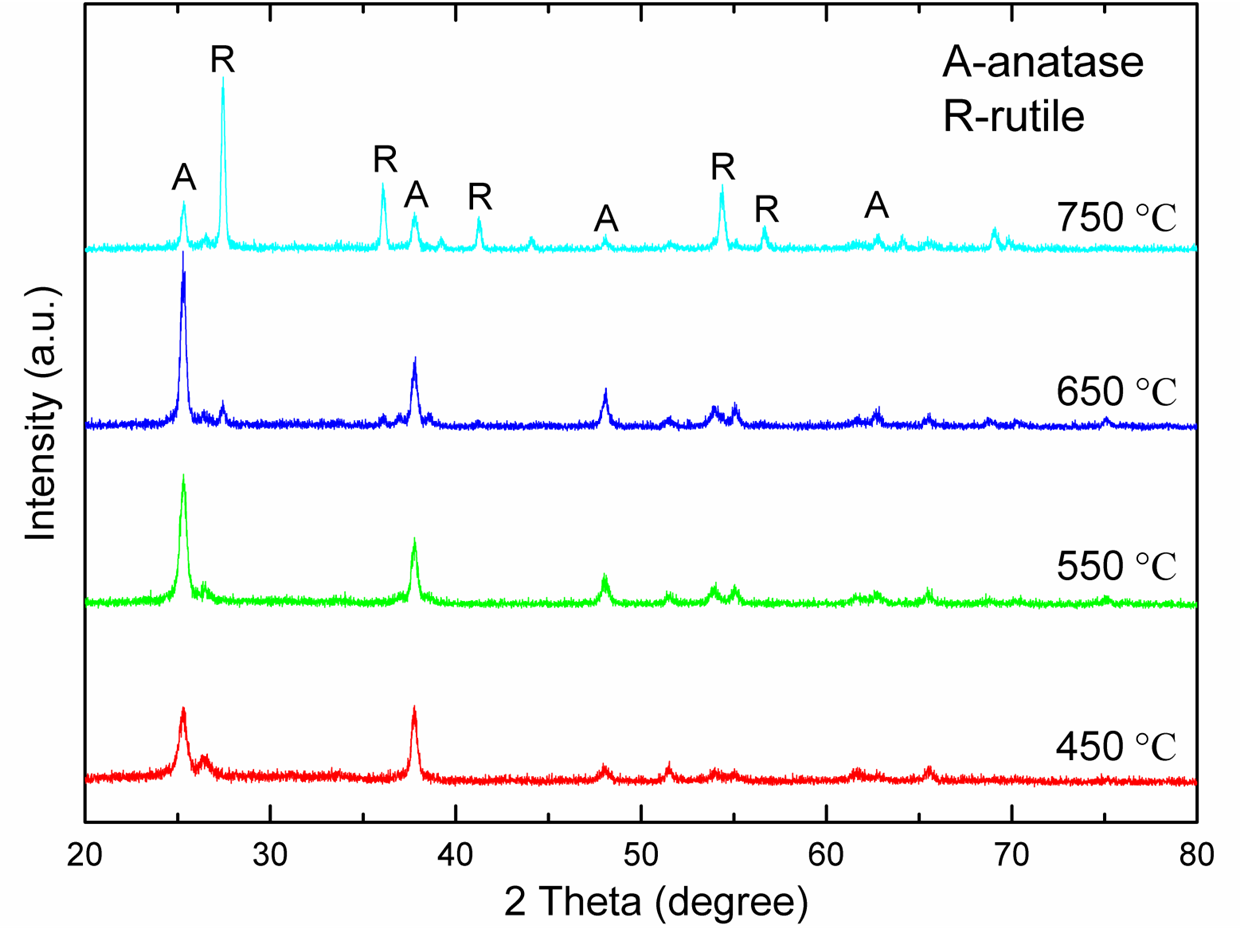


**Figure S3** XRD patterns of NT samples obtained by sonication in water.

The inner shells of NTs sonicated in water were consisted with small nanocrystallites, which were inclined to growth and aggregation by high temperature heat treatment. The sintering of NTs was accelerated, quickly reaching the critical crystallite size for anatase to rutile transition [1].

**References:**

1. Charette K, Zhu J, Salley SO, Ng KS, Deng D: **Gram-scale synthesis of high-temperature (900 °C) stable anatase TiO_2_ nanostructures assembled by tunable building subunits for safer lithium ion batteries.** *RSC Adv* 2014, **4:**2557–2562.
